# Accelerometry for remote monitoring of physical activity in amyotrophic lateral sclerosis: a longitudinal cohort study

**DOI:** 10.1007/s00415-019-09427-5

**Published:** 2019-06-11

**Authors:** Ruben P. A. van Eijk, Jaap N. E. Bakers, Tommy M. Bunte, Arianne J. de Fockert, Marinus J. C. Eijkemans, Leonard H. van den Berg

**Affiliations:** 1 Department of Neurology, Brain Center Rudolf Magnus, University Medical Center Utrecht, Heidelberglaan 100, 3584 CX Utrecht, The Netherlands; 2 Biostatistics and Research Support, Julius Center for Health Sciences and Primary Care, University Medical Center Utrecht, Utrecht, The Netherlands; 3 Department of Rehabilitation, Brain Center Rudolf Magnus, University Medical Center Utrecht, Utrecht, The Netherlands

**Keywords:** Amyotrophic lateral sclerosis, Accelerometry, Clinical trial, Longitudinal cohort study

## Abstract

**Background:**

The extensive heterogeneity between patients with amyotrophic lateral sclerosis (ALS) complicates the quantification of disease progression. In this study, we determine the value of remote, accelerometer-based monitoring of physical activity in patients with ALS.

**Methods:**

This longitudinal cohort study was conducted in a home-based setting; all study materials were sent by mail. Patients wore the ActiGraph during waking hours for 7 days every 2–3 months and provided information regarding their daily functioning (ALSFRS-R). We defined four accelerometer-based endpoints that either reflect the average daily activity or quantify the patient’s physical capacity.

**Results:**

A total of 42 patients participated; the total valid monitoring period was 9288 h with a 93.0% adherence rate. At baseline, patients were active 27.9% (range 11.6–52.4%) of their time; this declined by 0.64% (95% 0.43–0.86, *p *< 0.001) per month. Accelerometer-based endpoints were strongly associated with the ALSFRS-R (*r* 0.78, 95% CI 0.63–0.92, *p* < 0.001), but showed less variability over time than the ALSFRS-R (coefficient of variation 0.64–0.81 vs. 1.06, respectively). Accelerometer-based endpoints could reduce sample size by 30.3% for 12-month trials and 44.6% for 18-month trials; for trials lasting less than 9 months, the ALSFRS-R resulted in smaller sample sizes.

**Conclusion:**

Accelerometry is an objective method for quantifying disease progression, which could obtain real-world insights in the patient’s physical functioning and may personalize the delivery of care. In addition, remote monitoring provides patients with the opportunity to participate in clinical trials from home, paving the way to a patient-centric clinical trial model.

## Introduction

The progressive and debilitating nature of amyotrophic lateral sclerosis (ALS) restricts patients from participating in clinical trials. Trial participation is burdensome due to the frequent clinical assessments, laboratory tests and hospital visits. Consequently, there is a selective enrollment of patients, which obscures the collection of safety and efficacy data on the majority of patients [4, 5, 19, 25]. In addition, the extensive heterogeneity between patients complicates the quantification of disease progression [25]. This affects the design of clinical trials and their ability to detect treatment responses.

Therefore, there is an increasing interest in remote monitoring of efficacy endpoints [17]. Remote monitoring maximizes the collection of information outside clinical visits and could make in-clinic visits superfluous. Numerous studies are revealing the feasibility of remote monitoring of cardiac [23], respiratory [27], neurological [29], physical or homeostatic parameters [1, 9, 14]. Giving patients the opportunity to participate in clinical trials from home is intriguing, especially for debilitating disorders such as ALS. As ALS leads to progressive functional loss, remote monitoring of physical activity (i.e., accelerometry) could be an inexpensive method to assess a patient’s progression rate objectively. Using remote markers of disease progression may reduce the overall trial burden and potentially allow more patients to participate in clinical trials.

Currently, there are no data regarding accelerometer-based monitoring in patients with ALS. It remains, therefore, unknown whether accelerometry can accurately reflect disease progression or whether it can improve current trial endpoints. In this study, we provide an initial step towards validating remote monitoring of disease progression in patients with ALS.

## Methods

### Study population and procedures

Patients were recruited from the Treatment Research Initiative to Cure ALS (TRICALS) database. The TRICALS database is a web-based international patient registry for patients with motor neuron disease (MND). The database holds approximately 300–350 active Dutch patients at any given time. For this study, all active TRICALS patients were approached by e-mail and invited to participate. Patients were required to have a diagnosis within the MND spectrum [i.e., ALS, progressive muscular atrophy (PMA) or primary lateral sclerosis (PLS)]; no additional eligibility criteria were applied. The study physician (R.P.A.v.E) reviewed the medical records for all participating patients to confirm their diagnosis and to classify patients into five prognostic groups according to the ENCALS survival model, as described elsewhere [25]. Subsequently, patients were sent the ActiGraph GT9X Link (ActiGraph LLC, Pensacola, FL), a small (0.5 × 3.5 × 1 cm), lightweight (14 g) tri-axial accelerometer. The ActiGraph was worn on the right hip in the anterior axillary line using a belt clip during waking hours for 7 days. It was initialized to collect data at a sampling rate of 30 Hz. In addition, patients were asked to keep a wear time log and to provide information regarding their daily functioning (revised ALS functional rating scale, ALSFRS-R), weight and mood (Hospital Anxiety and Depression Scale, HADS). All study materials were sent and returned by mail every 2–3 months for a maximum of seven measurements (T0–T6). This study was approved by the Medical Ethics Committee of the UMCU (16/606). All study participants gave written informed consent to be approached digitally for research purposes and consented to participate in this study.

### Accelerometer data

The ActiLife (version 6.13.3) software was used to extract the raw accelerometer data from the ActiGraph. The 30-Hz data were summarized in 10-s epochs with application of the low-frequency extension (LFE) algorithm. The LFE algorithm increases the sensitivity for capturing lower intensity activities (e.g., sleeping, studying or watching television), which was hypothesized to be of relevance for elderly and neurologically impaired patients [8, 12]. Raw accelerometer files were processed to remove the recorded activity during mail transportation and to identify non-wear periods. Non-wear periods were identified using the non-wear time classification algorithm reported by Choi et al. [6]. Due to extremely low activity levels of far progressed patients (e.g., ALSFRS-R < 15), we defined a non-wear period conservatively as a consecutive period of no activity for 150 min. Finally, we calculated per day the total wear time in hours. To obtain an accurate estimate of the mean activity during a day, days with less than 8 h of total wear time were excluded from the analysis.

### Accelerometer-based outcomes

**Fig. 1:**
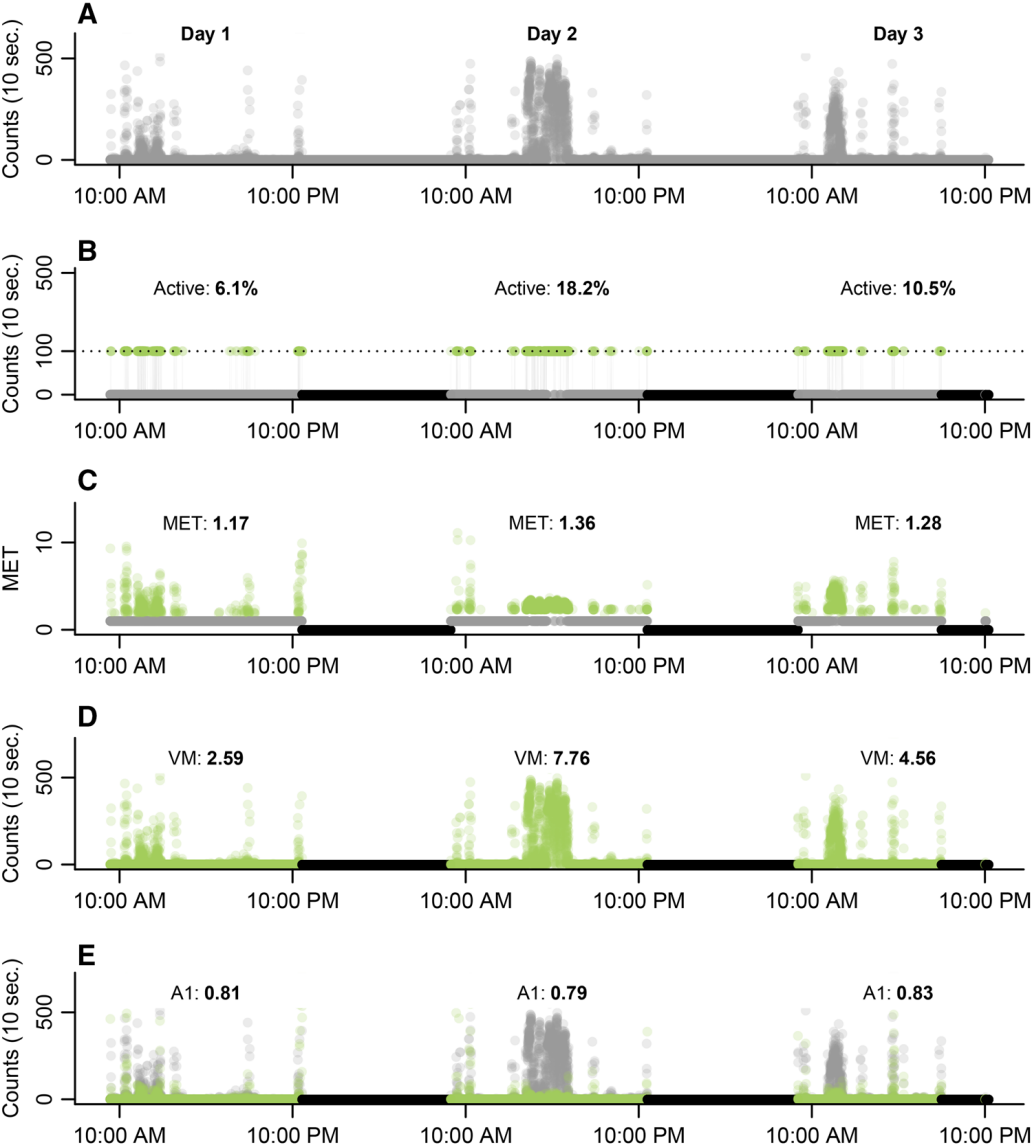
Raw accelerometer data of a single measurement and illustration of outcomes. Non-wear periods (black) were identified in raw accelerometer data (**a**). For the wear periods, we defined four outcomes: (1) %active, (2) metabolic equivalent (MET), (3) vector magnitude (VM) and (4) A1. **b** %Active; the activity count (*y*-axis) was split based on a 100 counts per minute cut-off and we calculated the proportion of being active (i.e., > 100 counts, green) [16]. **c** MET; the activity counts were recoded to MET (gray=MET 1) and averaged. When a patient is inactive (i.e., lying), the MET is 1 (gray) [7]. **d** VM; the average daily activity count (mean; mu) was multiplied by the daily variation in activity counts (*sd* standard deviation). **e** A1; instead of using the composite of three accelerometer axis (gray), we extracted only the vertical axis (i.e., movement against gravity; A1, green). The A1 was defined as the daily variation in the vertical axis (sd). The four outcomes resulted in different daily summaries with important differences in day-to-day variation. For example, the %active ranges from 6.1 to 18.2%, whereas the A1 ranges only from 0.79 to 0.83. This could have important consequences for the sensitivity to detect differential disease progression

The summarized and processed accelerometer data consist of approximately 10,000–30,000 observations per measurement (Fig. 1a). Observations are expressed as activity counts per 10 s. An activity count is based on the vector magnitude, i.e., the squared sum of the tri-axial data: $\sqrt {x^{2} + y^{2} + z^{2} }$, where *x*, *y*, and *z* are the vertical, forward and sideway axes, respectively. Due to the extent of the data, we defined four different outcomes to summarize daily activity into a single value: (1) %active; (2) MET score; (3) daily VM and (4) daily A1 (Fig. 1b–e). For the %active, we estimated the proportion of the vector magnitude counts that exceeded the 100 counts per minute threshold (Fig. 1b), which is consistent with more than sedentary activity (sedentary < 100, light < 760, moderate-to-vigorous < 2020 and vigorous ≥ 2020 counts per minute) [16]. For the second outcome, we translated the vector magnitude counts to Metabolic Equivalent of Task estimates (MET [7]), which were summarized by calculating the average daily MET score (Fig. 1c). Both summary statistics, %active and MET score, are reflecting the average daily activity of a patient, i.e., what a patient does during the day. They do, however, not directly indicate what a patient physically can do. This additional information can be partially extracted from the variation in vector magnitude counts. If the variation in vector magnitude counts is large, but the average vector magnitude small, this indicates that the patient is physically capable of making strong (i.e., high vector magnitude) movements, but chooses not to (e.g., due to a lack of motivation or fatigue). We defined, therefore, an outcome based on the average daily vector magnitude count and its variation (daily VM, i.e., average × standard deviation of the vector magnitude, Fig. 1d). The daily VM was estimated on the log (ln) scale due to its (zero-inflated) Poisson distribution. Similar to the daily VM, we evaluated a fourth outcome solely based on the variation in vertical axis (i.e., movement against gravity, *y*-axis), hereafter referred to as daily A1 (Fig. 1e). From Fig. 1, it becomes clear that each endpoint results in different daily summaries with important differences in day-to-day variation. For example, the %actives ranges in this illustration between 6.2 and 18.1%, while the daily A1 ranges only between 0.79 and 0.83. This could have important consequences for the sensitivity of each end point.

### Sample size calculation

At the time of study initiation (Q4 2016), no data were available for accelerometer-based outcomes in patients with MND. We hypothesized that physical activity was strongly correlated with the functional status measured by the ALSFRS-R. The rate of decline and nuisance parameters (i.e., variance–covariance matrix) was, therefore, based on the longitudinal patterns of the ALSFRS-R total score using data from the LITRA study [26]. The longitudinal sample size calculation assumed, conservatively, a 12-month follow-up period with quarterly measurements. In total, 34 patients were needed to detect 0.60 points per month decline in ALSFRS-R (i.e., the lower 25th percentile of individual ALSFRS-R slopes) with 90% power and an alpha of 5% [24]. With an expected 10–20% attrition rate per year due to death or study withdrawal, we recruited 42 patients.

### Statistical analysis

The primary aim of the analysis was to assess the longitudinal rates of decline in daily activity or disease progression. Linear mixed-effects (LME) models were used to estimate the mean population rate of decline; all LME models were fitted with a fixed effect for time and a random intercept and slope for time per individual. To quantify the heterogeneity in rates of decline between individuals, we calculated the coefficient of variation (CoV) per outcome. The CoV was defined as the variation in slopes (i.e., random effect of time) divided by the mean rate of decline. Similar LME models were used to assess the longitudinal correlation between physical (accelerometer) activity and clinical markers of disease severity (i.e., ALSFRS-R or King stage, as estimated from the ALSFRS-R) [2, 24]. We used standardized outcomes in the LME models to express the longitudinal associations as correlation coefficients with a similar interpretation as Pearson’s *r*. Finally, we evaluated the effect of each endpoint on trial design based on longitudinal sample size calculations as described in more detail elsewhere [15, 24]. LME models were fitted using the R *lmer* function (lme4, version 1.1-18-1) [3]. The R Physical Activity library (version 0.2–2, 2018) was used to process the raw accelerometer data [6]. Results were considered significant when alpha was less than 0.05.

## Results

### Patient population and feasibility

Between the 7th of October 2016 and the 1st of November 2018, 42 Dutch patients participated in this prospective longitudinal cohort study; their baseline characteristics are given in Table 1. Despite the lack of eligibility criteria, the study population consisted primarily of patients with a relatively good prognosis [28]. The total follow-up time was 503.2 months; on average, each patient was observed for 12.0 months (interquartile range from 5.9 to 18.1) and produced an average of 4.9 measurements. A total of 15 patients died during follow-up (overall 18-month survival 71.5%, CI 58.4–87.4%). Patients rated the burden to wear the ActiGraph on a scale of 0–10, where 0 indicates no burden, as low: mean 1.3 (95% CI 0.7–1.9, range: 0–7). Three patients rated the burden ≥ 5: two patients were afraid to lose the ActiGraph, whereas one female patient reported limited clothing options (e.g., could not wear a dress). Overall, the burden was similar for males and females (*p* = 0.78). In total, 694 valid ActiGraph wear time days were available for analysis with a total monitoring period of 9288 h and a mean daily monitoring time of 13.4 h/day. The wear time adherence of 93.0% was excellent (694 ≥ 8-h periods out of the 746 days).

**Table 1:** Characteristics of the patients at baseline

Characteristics	Overall (*n* = 42)
Age, mean (SD), (years)	60 (12)
Males, no. (%)	31 (74)
MND subtype, no. (%)	
ALS	39 (93)
PMA	3 (7)
PLS	0 (0)
Bulbar onset, no. (%)	7 (17)
Symptom duration (months)	
Median	25
Range	7–218
Diagnostic delay (months)	
Median	8
Range	2–130
Riluzole use, no. (%)	30 (75)
Body mass index, mean (SD), (kg/m^2^)	25 (3)
ALSFRS-R total score, mean (SD)	36 (8)
ΔFRS (points per month)	
Median	0.34
Range	0.05–1.24
Prognostic subgroup, no. (%)	
Very long	16 (38)
Long	14 (33)
Intermediate	11 (26)
Short	1 (2)
Very short	0 (0)

*MND* motor neuron disease, *ALS* amyotrophic lateral sclerosis, *PMA* progressive muscular atrophy, *ALSFRS-R* revised ALS functional rating scale, *ΔFRS* 48—ALSFRS-R score/disease duration [11]

### Longitudinal change in physical activity and daily functioning

Based on accelerometer data at baseline, patients were active or non-sedentary 27.9% (95% CI 24.8–31.1%) of the time (activity count > 100 per minute) [16], with a between-patient variability in baseline activity ranging from 11.6 to 52.4%. Table 2 provides the baseline and longitudinal monthly rates of change (i.e., slope) for the ALSFRS-R and accelerometer-based outcomes. All outcomes exhibited a strong declining trend over time (all *p* values < 0.001). The average monthly decline in ALSFRS-R was 0.59 points (95% 0.39–0.80); the average monthly decline in being active was 0.64% (95% 0.43–0.86). In all outcomes, there was between-patient variability in the rate of decline (i.e., the presence of both fast- and slow-progressing patients, all *p* values < 0.001). The between-patient variability, expressed as coefficient of variation (CoV), was lower in accelerometer-based outcomes than the ALSFRS-R; range 0.64–0.81 vs. 1.06. A lower CoV could positively affect sample size calculations and increase the sensitivity to detect differential disease progression.

**Table 2:** Longitudinal rates of change during follow-up

Outcome	Model parameters				Coefficient of variation
	Intercept	Slope^a^	95% CI^b^	*p* values^b^	
ALSFRS-R					
Total score	36.4	− 0.59	− 0.80 to − 0.39	< 0.001	1.06
Bulbar score	10.2	− 0.13	− 0.19 to − 0.07	< 0.001	1.34
Motor score	15.2	− 0.44	− 0.60 to − 0.28	< 0.001	1.10
Respiratory score	11.2	− 0.09	− 0.15 to − 0.03	0.006	2.02
ActiGraph					
%Active	27.9	− 0.64	− 0.86 to − 0.43	< 0.001	0.81
MET	1.71	− 0.018	− 0.024 to − 0.013	< 0.001	0.64
Vector magnitude (VM)	8.55	− 0.19	− 0.25 to − 0.14	< 0.001	0.77
Vertical axis (A1)	1.65	− 0.029	− 0.038 to − 0.021	< 0.001	0.74

Coefficient of variation (CoV) = between-patient standard deviation of slope/mean rate of change, a lower value indicates that there is less variation among patients and disease progression can be detected more accurately; *CI* confidence interval, *ALSFRS-R* revised ALS functional rating scale, *MET* metabolic equivalent. Linear mixed models were used to estimate the mean rate of change, CI, *p* value and CoV

^a^Slope is the mean monthly rate of change during follow-up

^b^95% CI and *p* value of slope (indicating whether the rate of change is different from zero)

### Correlation with disease progression

**Fig. 2:**
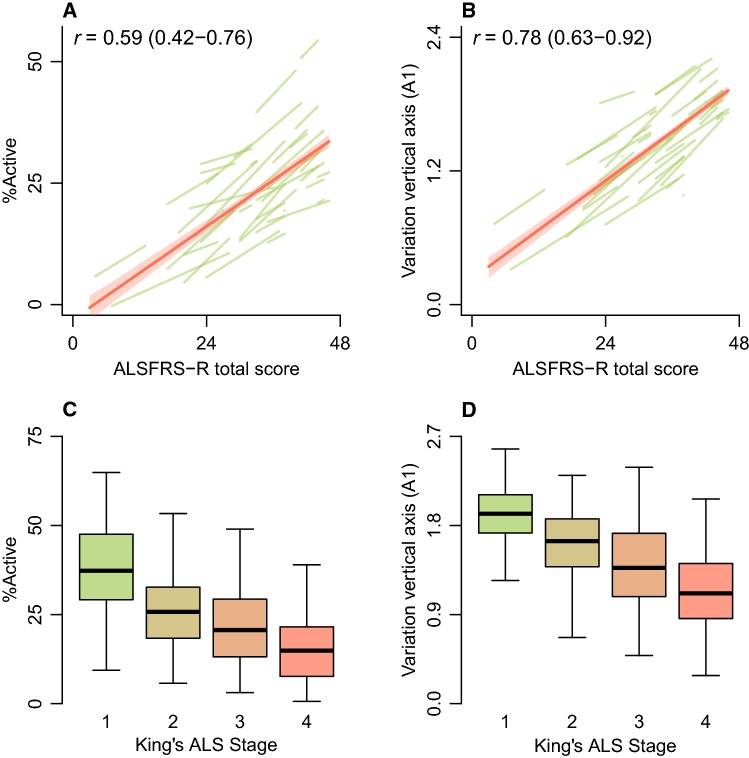
Correlation between accelerometer-based outcomes and disease progression. Longitudinal correlation between two accelerometer-based outcomes, %active (**a**) and variation in the vertical axis (A1, **b**), with the ALSFRS-R; *r* = Pearson correlation. The green lines represent the individual patient correlations. **c**, **d** Distribution of %active and A1 within clinical stages defined by the King’s ALS staging algorithm [2]

Figure 2a, b reveals the correlation between the ALSFRS-R and accelerometer-based outcomes. The MET score had the lowest correlation with ALSFRS-R (*r* 0.57; 95% CI 0.43–0.71, *p* < 0.001), whereas the variation in vertical axis (i.e., movement against gravity; A1) had the strongest correlation (*r* 0.78, 95% CI 0.63–0.92, *p* < 0.001); the correlation for daily VM was *r* 0.75 (95% CI 0.59–0.92, *p* < 0.001). The motor domain (i.e., ALSFRS-R item 4–9) was the primary driver of the correlation between A1 and the ALSFRS-R (*r* 0.83; correlation bulbar, fine motor, gross motor and respiratory domains were *r* 0.69, *r* 0.75, *r* 0.74 and *r* 0.55, respectively). A similar association was observed with clinical stage as defined by the King’s ALS staging algorithm (Fig. 2c, d, *p* < 0.001) [2]. Mean A1 levels were 1.82 (95% CI 1.70–1.94) for stage 1, 1.62 (95% CI 1.51–1.72) for stage 2, 1.40 (95% CI 1.30–1.51) for stage 3 and 1.12 (95% CI 1.00–1.24) for stage 4, suggesting a near linear trend across King’s ALS stages.

### Consequences for clinical trial design

**Fig. 3:**
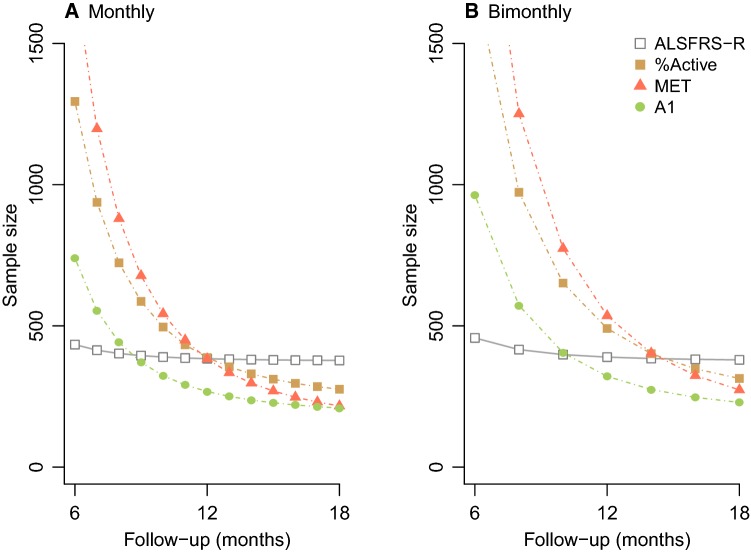
Longitudinal sample size calculations for three accelerometer-based outcomes. Models from Table 2 were used for the sample size calculation to detect a 25% reduction in slope with 90% power (per group). We evaluated different scenarios by varying the follow-up duration (*x*-axis) and using either a monthly (**a**) or bimonthly (**b**) sampling interval. The colors represent the different accelerometer-based endpoints. A1 = variation in vertical axis (i.e., movement against gravity). The sample size calculations are based on the observed slopes in Table 2 and do not account for missing data [15]. It is important to note that in other settings, the absolute sample size varies, but is unlikely to affect the relative differences between outcomes (i.e., absolute sample size are high in this example due to the relatively slow rate of progression of the enrolled population)

Finally, for each outcome, we explored the required group size to detect a 25% improvement in the rate of decline with 90% power for various follow-up periods and sampling frequencies (Table 2). When a monthly sampling interval is used, the accelerometer-based daily VM and A1 outcome outperform the ALSFRS-R when follow-up duration exceeds 9 months. At 12 months, a 30.3% reduction in sample size is achieved, which increases to 44.6% after 18 months (Fig. 3a). When a bimonthly sampling interval is used, the ALSFRS-R is outperformed after 12 months, resulting in a 17.5% reduction at 12 months, which increases to 39.7% after 18 months (Fig. 3b). The daily VM and A1 were superior outcomes in all settings when compared to the %active and MET. The difference between VM and A1 was minimal (5–7% difference in sample size).

## Discussion

In this study, we show the feasibility and value of remote, accelerometer-based monitoring of disease progression in patients with ALS. Accelerometer-based outcomes accurately assess the patients’ activity level under free-living conditions and provide an objective quantification of the disease progression rate across the disease spectrum. Accelerometry has a high adherence rate and may potentially lead to improvements in clinical trial design, not only by reducing sample sizes, but also by providing patients with the opportunity to participate in clinical trials from home. Reducing the number of hospital visits lowers the burden of trial participation and may better fit the physical abilities of patients with ALS. In the end, remote monitoring of disease progression may potentially increase the number of eligible patients, enhance enrollment rates and improve protocol adherence in ALS clinical trials.

The use of accelerometers, biosensors and medication adherence monitors is receiving increasing interest across all fields of medicine [17]. Digital heath monitoring could increase the efficiency of clinical trials, reduce costs, better reflect patient functioning and evaluate treatment responses in real-world settings [10, 13, 22]. Most importantly, remote monitoring of trial participants maximizes the collection of information outside clinical visits and could make in-clinic visits superfluous. This will pave the way to a patient-centric clinical trial model, where the trial is designed around the patient rather than fitting the patient into a clinical infrastructure. Despite these clear advantages, digital biomarkers are not frequently implemented in pivotal clinical trials [17]. Apart from the data complexity and potential ethical limitations [10], regulatory hurdles may be the main driver of their delayed utilization [13]. The limited standardization of the data capture, auditability and use for digital biomarkers may result in a lower level of consistency and quality compared to in-clinic measured endpoints. To overcome these hurdles, it is imperative to obtain insight into longitudinal patterns and confirm that digital biomarkers are valid surrogates for classical endpoints [13, 17].

Interestingly, we found a considerable degree of variation between the four methods to summarize daily activity and their ability to detect treatment responses (Fig. 3). Our results indicate that the daily VM and A1 are more suitable as accelerometer-based endpoints for clinical trials compared to the %active and MET. An important consideration is to distinguish between accelerometer-based outcomes that reflect what a patient does during the day (i.e., the percentage being active or the mean accelerometer count) with those that reflect what a patient can do (i.e., the variation in accelerometer counts like the A1). Although there is a decline in average daily activity as ALS progresses, the endpoints are affected by intrinsic patient-level characteristics, such as culture, life-style and motivation. Similar to a control population [16], there is a wide variability between patients in the amount of physical activity. By estimating the variability in daily physical activity, one can obtain an estimate of the range of activities a patient is capable of. Our results indicate that end points based on the variation in daily activity levels (e.g., VM or A1) have reduced between-patient variability and an increased sensitivity to detect differential disease progression.

Our study provides an important step towards validating remote monitoring of disease progression in patients with ALS. ALS is pathognomonic for the loss of motor neurons and the accompanying loss of muscle strength and function. The extensive heterogeneity between patients complicates the quantification of disease progression. This affects the design of clinical trials and their ability to detect treatment responses. In addition, the most common marker of disease progression, the ALSFRS-R, is affected by multidimensionality, which may prevent a sensitive assessment of the disease progression rate. Our results indicate that accelerometer-based outcomes approximate the ALSFRS-R, but have considerably less between-patient variability over time. This increases the sensitivity to detect treatment responses and may potentially lead to reductions in sample size and costs for mid- to long-term trials.

Our study has several limitations that should be considered. Similar to the white coat syndrome [18], activity monitoring might be affected by the Hawthorne effect (i.e., an alteration of behavior due to the awareness of being observed) [21]. This effect is problematic if one is interested in the average daily activity, but unlikely to bias the estimated progression rate. Our results indicate that high frequent or continuous monitoring may further increase the sensitivity of accelerometer-based outcomes. In addition, we only used 0.01% of the available data and a (Bayesian) modeling approach to define the entire dataset may significantly improve outcomes. Interestingly, when comparing the VM (three axes) and the A1 (only vertical axis), the vertical axis (i.e., anti-gravity movement) seems to be the most important axis for quantifying the rate of disease progression. There was no clear benefit of incorporating additional information from the forward and sideway axes (i.e., rotatory or sideways movements). This may be an important observation, as this may indicate that these axes hold limited information and may inflate the noise in the data. Moreover, and despite the strong correlations with functional measurements observed in our study, it remains essential to validate accelerometry prospectively with other key efficacy endpoints in ALS clinical trials, such as survival, muscle strength and lung function.

As a final note, ALS disease progression is not solely defined by a loss of (gross) physical activity, but also involves progression in domains such as bulbar, fine motor, respiratory and cognitive functioning. To fully quantify ALS disease progression there is, therefore, a need to objectively assess multiple domains in ALS, e.g., using speech analysis (e.g., from Aural Analytics) [20], apps evaluating fine motor tasks, actigraphy for gross motor functioning, and remote devices for body composition (e.g., percentage fat and fat-free mass) or pulmonary function. Besides the value of digital technology for clinical trials, the implementation of these technologies could also significantly benefit the delivery of care. Clinical decision algorithms based on remote data sources may be integrated into current healthcare systems to personalize clinical visiting schemes, or to optimize the detection (or prediction) of events such as respiratory failure or wheelchair dependency. In the end, these developments could significantly add to current methods of rating disease progression such as the ALSFRS-R.

In conclusion, in this study, we show the feasibility and value of remote monitoring of disease progression in patients with ALS. Accelerometry provides a non-invasive, remote and objective method for quantifying disease progression and correlates strongly with current outcomes. Accelerometer-based outcomes have the potential to be used as efficacy endpoint and may improve the efficiency of clinical trials. Remote monitoring provides patients with the opportunity to participate in clinical trials from home, paving the road to a patient-centric clinical trial model.
